# Consumers’ Attitudes Towards Functional Foods in the North-West Region of Romania (Transylvania)

**DOI:** 10.3390/foods15162861

**Published:** 2026-08-17

**Authors:** Dana C. Chira, Felix H. Arion, Mioara Mihăilă, Ioana A. Roman, Raluca A. Roman, Călin S. Vac

**Affiliations:** 1 Faculty of Horticulture and Business in Rural Development, University of Agricultural Sciences and Veterinary Medicine of Cluj-Napoca, 400372 Cluj-Napoca, Romania; dana-carmen.chira@usamvcluj.ro (D.C.C.); calin.vac@usamvcluj.ro (C.S.V.); 2 Faculty of Agriculture, University of Life Sciences “Ion Ionescu de la Brad” Iași, 700490 Iași, Romania; 3 Transversal Competences Department, University of Agricultural Sciences and Veterinary Medicine of Cluj-Napoca, 400372 Cluj-Napoca, Romania; ioana.roman@usamvcluj.ro; 4 Faculty of Chemistry and Chemical Engineering, Babes-Bolyai University, 400006 Cluj-Napoca, Romania; alina.roman1@ubbcluj.ro

**Keywords:** functional food, consumer behavior, nutritional information, entrepreneurship in health, consumer health awareness, health benefits

## Abstract

Consumers have become more interested in leading better and higher quality lives, and have become more aware of the health effects nutrition has on their body, stimulating the development of functional foods (FF), recognized for their ability to provide physiological benefits that go beyond conventional nutritional function. The evolution of this product category is supported by the progress of research on bioactive compounds and changing consumer preferences, but empirical evidence regarding the level of knowledge, acceptance and purchasing behavior of Romanian consumers remains insufficient. Meanwhile, technology in the food industry has evolved significantly in recent decades, creating opportunities for entrepreneurs and consumers. Using empirical evidence from a survey carried out on the consumers in the region of Transylvania, the study uses a cross-sectional observational design and investigates the awareness of and the population’s differences in perception and attitude on FF products depending on the generations and socio-economic situations consumers belong to, and the impact FF have on the market. The analysis focuses on food choice behaviors, degree of trust in health benefits and factors influencing the decision-making process. The results show that consumers are not aware of the FF concept, but understand the relation between food choice behaviors and personal health and act accordingly. Results also show that older consumers are more aware of their health, and younger consumers are more influenced by trends. This paper can be of importance to consumers, entrepreneurs, food and technological industries, governments and universities.

## 1. Introduction

Contemporary society, also known as, especially in developed countries, consumer society, is increasingly met with challenges that are directly related to the issue of consumed products and consumer behavior. In a world that has become emancipated and evolved on the steps of the ladder of needs, consumers are proving to be more and more interested in enjoying the best possible life, based on comfort and quality. Added to these expected benefits is the growing awareness of the effects of food consumption on health.

Even though there is no clear definition yet, functional foods (FF) are complex products that are conceptualized at the intersection of food science, nutrition and public health. It can also be easy to confuse this category with conventional food products, or even nutritional supplements, or organic foods. To focus the analysis on a clear area, the paper only covers functional foods. Few but critical features make the difference in everything from consumer items to health benefits or health claims [1]. “Functional foods are those that can be consumed within the normal diet and that contain biologically active compounds, with the potential to improve health or reduce the risk of disease.” [2]. This perspective has been expanded by research on the role of bioactive compounds in metabolic, inflammatory and oxidative processes, as well as in supporting cardiovascular, digestive and immune functions [3,4,5,6,7].

The Western culture’s focus on creating a better and high-quality life has increased significantly in the last 20–25 years, which has generated major and multiple changes in consumer behavior, especially when it comes to food choices. A major reason for these changes is the recognition of the mutual influence between diet and health. The effects of food from daily nutrition have become an important factor in Western culture, due to the evolution from simple desires to survive, satisfy hunger and prevent disease, to the current desire to use food to improve well-being and health and to reduce the risk of diseases in the population. That said, society is shifting its focus from “adequate nutrition” to “optimal nutrition”, which is creating changes and development needs in nutritional science for new products that improve well-being, mentally and physically, and reduce the risk of disease [8].

The present work is an analysis of consumer perception regarding FF products. The scope of analysis is limited to consumers from the North-West Region of Romania (Transylvania). Specifically, the differences in perception and attitude that the analyzed population manifests according to the generation they belong to are investigated. Another essential aspect pursued by the work is the degree of awareness and perception of consumers in the analyzed region regarding FF products.

The predominantly quantitative research provides insights into how consumers make food choices and the factors behind those choices, as well as insights into healthy lifestyles. The research also focuses on analyzing how consumers’ perception of functional foods differs depending on the generation they belong to, with the consumers divided into the following generations: Baby Boomers, aged between 56 and 76; Generation X, aged between 41 and 55; Generation Y, also known as Millennials, between the ages of 26 and 40; and Generation Z, aged between 18 and 25 years.

With regard to this particularity of the research, i.e., the differential-comparative analysis across generations, it aims to find out how open each generation is to functional foods, along with the usual food choice behaviors and how much emphasis is placed on maintaining good health. In addition, the study will investigate how open the population is to functional foods, analyzing the potential willingness to purchase such products and the degree of trust in the benefits provided by healthy foods. Choice behavior specific to human consumption consists of a mental process that transforms perceptions of several options into a single choice. The range of choice behaviors is quite wide: intuitive, automatic and impulsive, and conscious-deliberate [9].

Trust, formed on the basis of solid and coherent cultural values, traditions and education, is a mental state of a person expressed in relation to another person or good, a product over which there is no possibility of control [10].

The present research describes the attitudes and purchasing-related perceptions of adults from the Transylvania region who participated in a non-probability online survey. Because convenience and snowball recruitment were used and the final sample is imbalanced by gender, education and generation, the sample should not be considered statistically representative of the Transylvanian population. Accordingly, the findings are interpreted as evidence for the characteristics and associations observed within the analyzed sample rather than as population estimates.

Essentially, the research investigates the current attitudes of Transylvanian consumers toward functional foods. Thus, the research scope is limited to people from the Transylvania region of Romania, a diversified and representative group.

### 1.1. The Concept of Functional Foods

The functional-food concept was first introduced in Japan, a country that consumes a lot of rice. “Fine Rice” is the first functional food approved by Food for Specified Health Use (FOSHU) [11]. Research shows that consumers do not consciously recognize the concept of FF, but intuitively act in the sense of attracting the benefits resulting from the interaction between the consumption of FF and the state of health. Consequently, consumers display a behavior based on the intuitive choosing of functional foods. The development of functional foods can be viewed as a progression from food primarily intended to satisfy hunger and provide adequate nutrition, to foods increasingly associated with safety, quality and disease prevention, and finally towards products formulated or selected for additional physiological or health-related benefits.

The development of functional foods is supported by both technological progress and changes in consumer behavior. Polyphenols, dietary fibers, polyunsaturated fatty acids, bioactive peptides, probiotics and prebiotics are associated with antioxidant, anti-inflammatory, immunomodulatory and cardioprotective effects, but the success of these products depends on how the benefits are understood, valued and integrated into the purchase decision [5,7,12,13]. Thus, the acceptance of functional foods is not determined exclusively by their nutritional value, but also by consumer trust, the relevance of the communicated benefit, previous experience and the compatibility of the product with current eating habits [14,15,16,17].

Regarding the differences by age category, it is observed that older consumers are much more aware of their health, at least compared to younger generations. In turn, children do not significantly influence their parents’ purchasing behavior, except in the case of high interest in health. The presence of differences between generations regarding the consumption of functional foods is noted, with direct dependence on the interests of each generation. Older generations prefer to consume products that improve the overall well-being of the body, while younger generations prefer to consume products that increase their energy and improve physical condition [18]. This difference in perception, attitude and consumption decision is analyzed in the present paper within the research population.

### 1.2. The Functional Foods Market

For a focused analysis from the perspective of the exchange of functional foods, starting from the interest of the producer and ending with the interest of the consumer, reference is made to the framework that marks this exchange, namely, the market of functional foods. This is constantly growing, although it is still considered an experimental environment [19]. Although the demand for such foods depends a lot on the culture and traditions of each country [20,21], one thing is certain: consumers are interested in consuming food products that are more suitable for maintaining good health. In order to develop the FF production environment, investments and detailed analyses are required, but there is no certainty of market success [22,23]. The functional foods market is driven by consumers, and successful products are created only by understanding consumer motivations and interests [24].

In order for FF products to reach the market without major efforts, the process of scientific research of the product is essential. To be successful, it is necessary to follow the route of an approach based on the advantages offered by the product: performing clinical studies, selecting a proprietary ingredient, and building a brand [25].

Even if the development of the FF market is not carried out at a rapid pace, an increase in market share is observed, primarily through the emergence of new economic opportunities, along with health benefits [26]. The success of functional foods is not only in the interest of producers (entrepreneurs), but also of consumers who are concerned about their health status and increasing the quality of life [27].

The functional foods market is consumer-driven, so understanding consumers’ motivations and interests is the key to success [24]. Studies show that the main trends driving the success of functional foods are age, gender, education, demographic changes, healthcare costs, media, access to information, nutrition labeling, development in food technology, brand differentiation, value for money, and changes in the perception of healthy diets and disease prevention [15,20,28]. These elements, along with consumers’ attitudes, affect the acceptability and intention to use functional foods [15,29,30].

A limiting aspect of the development of the FF market is given by the lack of a clear legal framework regarding functional foods, due to the fact that they are considered “hybrid” products, so they are not yet very eloquently defined [26].

### 1.3. Psychological Aspects in the Consumption of Functional Foods: Motivation, Preference, Attitude, Behavior

Food choice is the result of the interaction between cognitive, psychological, economic and socio-demographic factors. Education level, nutritional literacy, health concerns, credibility of health claims and price perception significantly influence the acceptance of functional foods [31,32,33,34]. At the same time, research on planned behavior and health behavioral models shows that purchase intention is built by relating anticipated benefits to perceived barriers, of which cost, accessibility and distrust in label information are particularly relevant [35,36,37].

Intergenerational processes provide a useful conceptual lens for functional foods because consumers born and socialized in different historical, technological and economic contexts may develop different health priorities, information-search habits, trust patterns and responses to food innovation. Generational Cohort Theory therefore treats generation as a cohort-level context rather than simply an age category, while the Theory of Planned Behavior and the Health Belief Model help explain how attitudes, perceived benefits, perceived barriers and social influences can translate into food-choice intentions [35,36]. In the present study, generation is consequently used as a segmentation variable through which differences in awareness, product-attribute evaluation, health perceptions and purchase-related behavior are explored. These differences should be interpreted cautiously because age, education, income and occupation may be correlated in a cross-sectional sample.

Recent research also demonstrates that generational segmentation can reveal meaningful differences in food perception and choice. Cabal-Prieto et al. compared Baby Boomers, Generation X, Y and Z in sensory and cognitive evaluation of artisan tortillas, while Uliano and Lerro identified generational differences in preferences and willingness to pay for antioxidant-rich pomegranates [38,39]. These studies support the value of an intergenerational perspective, but they concern different food products and markets. The present study extends this perspective to functional foods in a Romanian regional context, combining awareness of the FF concept with health perceptions, product attributes and purchase-related behavior.

Regarding the state of health consciousness, the predominant manifestation of the tendency to adopt a healthy lifestyle is noted; this generates more positive attitudes towards functional foods, and consequently an increase in their consumption [17,31]. In addition, the apparent link between beliefs about health benefits, perceived health status, and knowledge of food components is supported by consumer acceptance of functional foods [14,17].

But apart from the influencing factors strictly related to the functional characteristics of food, consumers are also strongly influenced by quality, packaging, price and brand [40]. In this way, a favorable perception of the health guarantee is given by the accepted taste and high quality of the product, the attractiveness of the packaging, the higher prices and the imposition of the brand, all of which outline a high level of trust in the products and, consequently, the purchase and consumption of functional foods [41]. Other elements considered in the research are: the preferential decision between healthier food products and consumer confidence in the health benefits of FF products. The analysis is supported by reported data showing that most consumers prefer to consume food products to improve their immune system and trust that consuming functional foods can improve their overall health.

Globally, consumers have become increasingly health-conscious by experiencing the consumption of products that influence their health. More than 56% of Europeans reported that consuming food and drink can improve their health [22]. In addition, consumers are increasingly interested in their health, which they monitor more and more carefully in order to maintain good health and prevent diseases [42,43,44]. This tends to lead to consumption of products with health maintenance claims [37,45,46,47]. Health consciousness and a healthier lifestyle have been shown to be drivers of an increasingly favorable attitude towards functional foods, along with the increasing consumption such foods [17,31]. Even without knowing the term functional food, the consumer is sensitive and attentive to messages about the health benefits that functional products have [24].

However, consumer acceptance of functional foods depends on the product category and promotion strategy [48]. A strong relationship between personal beliefs and the acceptance for consumption of functional foods is viewed from several perspectives: the perception of health claims, the contribution to disease prevention, and the benefits of food for health [14,49,50,51]. Functional foods may be perceived as healthier depending on consumers’ knowledge, familiarity with functional components, and trust in the health benefits of these products, all of which complicate understanding the decision-making process [17,52]. Thus, lack of knowledge of the content and benefits of functional foods contributes to a major discouragement of consumption [14,53].

### 1.4. The Functional Foods Situation in Romania

In Romania, examples of commonly encountered functional-food categories include fermented dairy products containing probiotics, fiber-enriched cereals and bakery products, plant-based products rich in polyphenols, and foods or beverages marketed with permitted nutrition or health claims [54,55,56,57,58]. These examples illustrate that functional foods are not necessarily a separate food category in everyday consumption; rather, functionality may be associated with specific ingredients, formulations or authorized claims.

Romania follows the broader European trend towards food choices associated with health and prevention, but empirical research specifically linking knowledge of the FF concept, perceived benefits, purchasing behavior and socio-demographic characteristics remains limited. The term “gap” in this study therefore refers to a specific empirical gap: the scarcity of integrated, regionally focused evidence on how Romanian consumers across different generational cohorts understand and evaluate functional foods. Existing European research has examined functional-food acceptance, health consciousness and purchasing determinants [17,31,34], while more recent studies have demonstrated generational differences for specific food products [38,39]. The present study differs by examining these dimensions jointly in a Romanian regional sample and by explicitly comparing four generational cohorts within the same survey instrument. The contribution is therefore contextual and empirical rather than a claim of national representativeness.

This gap is important in the context in which cardiovascular diseases continue to represent the main cause of mortality in Romania, and national health strategies explicitly promote the adoption of preventive dietary behaviors and the development of interventions based on nutritional education [59,60]. Assessing how consumers understand, perceive and use functional foods can provide useful information for designing communication strategies, adapting products to market requirements and underpinning public policies aimed at preventing chronic diseases.

The regulatory context is also important for interpreting functional-food communication in Romania. As an EU Member State, Romania applies the EU framework governing nutrition and health claims, including Regulation (EC) No 1924/2006, which requires nutrition and health claims used in food labeling and advertising to comply with specified conditions and scientific substantiation. Where products or ingredients fall within the novel-food scope, Regulation (EU) 2015/2283 provides the relevant authorization framework. General food information and labeling requirements are further governed by Regulation (EU) No 1169/2011. Thus, consumer communication about functional foods should distinguish between a food’s nutritional composition, an authorized health claim and broader marketing language [61,62,63].

Based on these premises, the research aims to assess the level of knowledge of the concept of FF, analyze perceptions and purchasing behavior, and identify the influence of socio-demographic characteristics on acceptance-related indicators among adult survey participants from Transylvania. The specific contribution is to integrate cognitive (awareness), attitudinal (health perceptions and trust), behavioral (purchase experience and intention) and product-attribute dimensions within an intergenerational analysis of a Romanian regional sample. The study does not seek to estimate population prevalence or to establish causal effects.

The research objectives and hypotheses are formulated as follows. O1: To analyze functional-food choice behaviors and the product attributes considered in purchase decisions. H1: The importance attributed to selected functional-food attributes differs across generational cohorts. O2: To analyze consumer confidence in the health benefits of functional foods. H2: Perceptions of health benefits and disease-prevention potential differ across generational cohorts and socio-demographic groups. O3: To analyze the role of functional-product factors—packaging, taste, brand, scientific certifications and nutritional information—in purchase-related evaluation. H3: Selected socio-demographic characteristics, particularly generation and education, are associated with differences in the evaluation of these product factors. Because the design is observational and cross-sectional, these hypotheses are tested as hypotheses of association, not causality.

## 2. Materials and Methods

### 2.1. Analysis Parameters of Functional Foods in Purchase and Consumption Decisions

Essentially, when making a purchase, consumers look for cues about quality, price, packaging and labeling, along with attractiveness, credibility and uniqueness [40,41,64,65]. It is therefore believed that consumers need to be educated about functional foods, as not all ingredients are familiar to them, and proper labeling is a solution to this. About 90% of consumers check food labels before buying something [40,66,67]. Research also shows that, almost exclusively, consumers do not know the composition of a food product, but only perceive it as healthy or not [68,69,70,71,72].

*The packaging*. Visual cues have become more important than informational cues in guiding food consumption decisions, meaning that images and colors are more important than label information [73,74]. This aspect is reflected by the packaging method, which turns out to be one of the most important factors in the purchase and consumption decision [75]. With 73% of purchase decisions resulting from packaging appreciation, it proves to be a “silent seller” [76,77,78]. Thus, the way the product is presented must attract the attention of consumers and inspire their purchase and consumption decision [79]. The effectiveness of a message is supported by the number of senses stimulated, and packaging has the ability to influence flavor perception and ultimately food acceptability [80].

*Taste* is an essential attribute for the presentation and success of a food product, and consumers are not willing to buy functional foods with inferior taste [14,15,19,27,45,81,82]. “Taste is the most specific meaning that people have” and it is difficult to associate it in a general way with consumer preferences, because some people like a certain taste while others do not like [83]. Despite the health benefits or the acceptable purchase costs, the sensory properties of food become a priority, and for a product to be successful in the market, it should agree with and be accepted by consumers [84,85,86]. Regarding functional foods, it appears that consumers are not willing to compromise taste for health benefits [14]. Taste remains a critical attribute in all consumer groups, an aspect that is very important in the purchase and consumption decision, especially when done repetitively.

Regarding *gender*, women are found to pay more attention to food labels compared to men [40]. Also, women represent the majority of FF consumers and have more interest in a healthy lifestyle [19,27,45,47,87], being more responsible regarding the consumption of certain foods, given their role as protector of the well-being of other family members [88].

*Brand* has an extremely strong influence on purchase behavior and studies show increased willingness to pay for well-known brands [89]. The choice of a particular brand can indicate or increase the social status of consumers [42,76]. Well-known brands are considered to have better taste, so they create positive attitudes and loyalty [84]. Familiarity with a brand gives consumers additional knowledge when making a choice, creating consistent behavior. Consumers do not pay as much attention to information on labels, but they recognize the brand [90]. Children and young people have a very strong connection with brands intrinsically, so they express a strong influence on parents’ purchasing behaviors and on the final food choice [91].

*Differences between generations*. Given that the consumption of functional foods contributes to disease prevention, middle-aged and older people should be more motivated in their consumption decision [14,27]. The individual factors that influence the decision to consume functional foods are: food preferences, culture and personal beliefs, the social environment of belonging, the economic situation, the geographical environment, etc. [92]. It has been found that, although consumption needs and habits become more diverse with age [45,93,94,95], elderly people remain skeptical about the consumption of functional foods [41,47]. Interestingly, younger consumers seem to be more willing to try new foods, so they have a more favorable attitude towards functional foods [41]. The older generation prefers foods that relieve and improve general physical condition and increase energy, while the younger generation is very interested in reducing cholesterol or strengthening the cardiovascular system [18,96,97]. As the younger generation is not interested in cooking much, they will rely on packaged foods [40,98,99]. Also, the willingness of this generation to pay more for food to ensure a healthy lifestyle is observed [100].

### 2.2. Methodology

Understanding the factors that influence consumer behavior contributes to the success of a business [101]. It is essential for a business to know the behavior of the consumer, to analyze why a good is purchased more than others, what determines the consumer’s decision to buy or not buy and what measures should be taken to draw the consumer’s attention to a product or service [102]. Recent research shows that ecological marketing factors (for clean products, such as functional foods) changing consumer behavior towards healthy products and favorable market conditions have a significant and positive impact on sustainable entrepreneurship [103]. Moreover, sustainable entrepreneurship is educated entrepreneurship, which, over time, brings greater satisfaction [104] and more important economic growth [105].

The general purpose of this research is to examine differences in perception and attitude towards functional foods across consumer generations and socio-economic groups, with particular attention to awareness, health-related perceptions and purchase-related behavior. The objectives are explicitly designated as O1–O3 so that the results and conclusions can be aligned with the analytical structure of the study.

- O1. Analysis of functional-food choice behaviors;
- O2. Analysis of consumer confidence in the health benefits of functional foods;
- O3. Analysis of functional-product factors—packaging, taste, brand, and scientific certifications.

A questionnaire-based market survey was used as the main data-collection method. Recruitment combined purposive stratification by age and socio-demographic characteristics with convenience and snowball approaches. The resulting sample is therefore a non-probability sample. These procedures were intended to obtain participation from all four age cohorts, but they cannot guarantee population representativeness. In particular, the final sample contains a higher proportion of women and highly educated respondents than would be expected in a population-representative sample.

The data-collection instrument was a structured questionnaire containing 17 closed questions, administered through Google Forms in May 2026 (Appendix A). It targeted consumers aged 18–76 from Transylvania and covered awareness of functional foods, previous purchase experience, product attributes, health-related perceptions, trust, purchase intention and seven socio-demographic characteristics. Participation was voluntary and anonymous, and completion implied informed consent. A total of 223 responses were recorded; 215 complete responses were retained for analysis. The eight excluded responses were incomplete and could not provide the complete set of variables required for the contingency-table analyses. No additional exclusion criterion was applied.

The structure of the survey was created in such a way as to investigate the main research problem and respond to the proposed objectives, to provide an appropriate context for processing the results and further analysis. The questionnaire was built on the basis of the following reference parameters: knowledge about FF products, purchasing behavior, consumer confidence and preference in the consumption of functional foods and their choices. Also, for the consideration of independent variables, questions about demographic data were inserted, which were very useful in segmenting the working sample.

The data was analyzed using descriptive statistics and Pearson χ^2^ tests in Statgraphics (Version 19). Absolute and relative frequencies were calculated for the investigated variables, and associations between socio-demographic variables and perception or behavior items were evaluated at *p* < 0.05. Because the sampling procedure was non-probabilistic, a conventional population margin of error is not reported; the previously stated 7% margin of error has therefore been removed. Cramér’s V was calculated for the contingency tables to quantify the magnitude of associations. The effect sizes were interpreted cautiously because several tables contain multiple categories and the subgroup sizes are unequal.

A sensitivity calculation following the reviewer-specified one-sample *t*-test formula pwr.t.test (*n* = , d = 0.8, sig.level = 0.05, type = “one.sample”, alternative = “two.sided”) gives power values of approximately 1.000 for Generation Z (*n* = 82), 1.000 for Generation Y (*n* = 52), 0.999 for Generation X (*n* = 43), and 0.998 for Baby Boomers (*n* = 38). These values are presented only as the requested sensitivity calculation: the formula is for a one-sample *t*-test and is not a direct power calculation for the Pearson χ^2^ contingency-table tests used here. Therefore, the study does not claim that the χ^2^ analyses are adequately powered for all subgroup comparisons, and the relatively small Baby Boomer subgroup remains a limitation.

To estimate the effect size, Cramér’s V coefficient was used, since the χ^2^ test indicates the existence of an association, but not its intensity. The values were interpreted as indicative according to the guidelines proposed by Cohen [106], taking into account the size of the contingency tables. The item regarding product categories, with multiple responses, was analyzed descriptively, because applying an omnibus test would have violated the hypothesis of independence of observations.

The processed demographic data shows the following profile of the sample: the majority is female, young people between the ages of 18 and 25, in or graduated university or working, with lower incomes. For the parameter knowledge of functional foods, most of the participants are not familiar with the concept of FF, even though most of them are active consumers without realizing it.

The analysis focused on food choice behavior in relation to purchase sources, importance of product-related elements, functional-food categories and situational factors influencing the final purchase decision.

In order for the respondents to understand all the questions and answer accordingly, sets of questions were structured by objective categories. The first two questions address knowledge of functional foods, followed by a brief explanation for consumers who are unfamiliar with the topic. In order to favor the reiteration of a possible previous contact with functional products, the second question was drafted, which aims to find out whether they have bought or not, up to the time of the survey, any functional food. The next four questions target consumer purchasing behavior by understanding the importance of product attributes in terms of packaging, taste, price, brand, scientific certifications, and nutritional information. The benefits that the products could have for health, as a remedy or prophylactic, are also considered. The next question concerns the trust in the effects of functional foods in terms of improving general health or preventing diseases and the future intention to purchase functional products. The last seven questions concern demographic data: age, gender, education, professional activity (occupation), income, marital status and family structure.

The questionnaire was intentionally concise to reduce respondent burden in an online market survey. The awareness construct was operationalized through recognition of the FF concept and previous purchase experience rather than through a multi-item psychometric scale. Accordingly, reliability coefficients such as Cronbach’s alpha are not applicable to the binary awareness item or to the heterogeneous single-item indicators. Future studies should use validated multi-item scales for functional-food knowledge, health consciousness and trust, and should assess internal consistency and construct validity before multivariate modeling.

## 3. Results

For the analysis of the collected data, they were organized in tables of frequencies with absolute and relative percentage sizes. The obtained results were processed to correlate the independent variables with the dependent variables in the decision to purchase functional foods.

Regarding previous experience with functional products, the majority of participants (88.37%) declare that they have previously purchased such products. Among those surveyed, 6.51% did not have previous contact with functional products, and 5.12% are not convinced of the type of products consumed. To highlight the importance of product attributes that can influence the final purchase decision, reference was made to packaging, taste, brand and scientific certification. The results are presented in Table 1 below, while the scale of importance of the attributes is detailed in Table 2.

As can be seen, the most important parameter appreciated by the surveyed consumers is the taste (72.09%), followed by the packaging with a fairly large margin (49%). Interestingly, the brand has the lowest importance in the final decision to buy functional foods (9.77%).

The analysis of consumption preferences and the trust that consumers have in functional foods is summarized below (Table 3) and the results obtained from the survey are presented. Next, Table 4 shows the results for the segmentation of groups based on demographic data. Reference was made to the following parameters:

- The contribution of functional foods to the prevention of a disease or for prophylactic consumption (for example, in the questionnaire, on a simple flu);
- Trust in the benefits of functional foods to improve general health;
- Trust in the benefits of functional foods to prevent some diseases.

Using Statgraphics, the Chi-Square test was performed to analyze a set of correlations between the main analysis variables. These led to understanding the links between age, education, family structure and gender with various elements included in the survey questions: awareness, packaging, taste, brand, certifications, nutritional information, trust directed towards impact on health and disease, preferences during flu seasons, and perceived trust for health assurance (Table 5).

Baby Boomers and Generation X are unfamiliar with the concept of FF, while Generations Y and Z appear to be more familiar. So, the younger generations are more aware of this food category, passing this knowledge on to the next generations. That is why it is considered that this concept of FF will become known to most consumers in the near future.

The influence that age can have on the importance of packaging in final purchase decision was analyzed starting from the null hypothesis that age does not influence the importance of the packaging that products have and the alternative hypothesis that it does. The results show that every age level values the importance of packaging. However, Millennials are more careful about packaging, and most participants from this group said it was important, compared to the rest of the generations who said it was somewhat important. Therefore, there is not always a significant influence in the decision-making process.

Regarding the influence that age can have on the importance of nutritional information in the final purchase decision, the null hypothesis was that it does not influence, and the alternative hypothesis that it influences. The result shows that age influences the importance of nutritional information. The data shows that all generations consider nutrition information at some point before making a purchase decision.

The influence of age on consumption preference during flu seasons is based on the null hypothesis that it does not influence it and the alternative hypothesis that it does. The result shows that, with a 95% confidence level, age influences consumption preference. Generation Z is more inclined to take supplements or do nothing rather than consume functional products during flu season. The other generations tend to consume functional products and trust the health benefits of their food choices.

Education was statistically associated with the reported importance of brand (χ^2^ = 38.331, df = 24, *p* = 0.0321; Cramér’s V = 0.211). In this sample, respondents with higher education reported greater regard for brand. This is an association within the observed sample and should not be interpreted as evidence that education causes greater or lower reliance on brands. This trend may reflect the composition of the sample and the distribution of other socio-demographic characteristics.

For scientific product certifications, education influences this component in final purchase decisions with a 95% confidence level, and consumers with higher levels of education consider the presence of certifications more important. Likewise, education influences consumer perception and consumer trust in functional products, with people with higher education having more confidence in these benefits. Regarding the perception and trust that functional foods can prevent diseases, the null hypothesis is that the level of education does not influence the perception, and the result shows that education influences the perception and trust of consumers. In addition, participants with higher levels of education have more confidence in functional foods, with small differences even between those with master’s and doctoral degrees. Education, however, does not influence what participants consume during flu seasons, so regardless of education level, consumer preferences do not change.

Education influences the importance of nutritional information with a confidence level of 95%. The data shows that all participants feel this is important, and those with higher levels of education tend to be more influenced (Table 6).

The test hypotheses for the parameters in Table 7 are as follows:

- The null hypothesis is that family structure does not influence awareness, health or the preference in consumption and the alternative hypothesis is that it does. The results show that family structure influences the importance of these components.
- The null hypothesis is that the family structure does not influence the importance of the package, taste or brand, and the alternative hypothesis is that it does. The results show that the family structure does not influence the importance of these components.
- The null hypothesis is that gender does not influence the importance of taste, and the alternative hypothesis is that it does. The results show that gender does not influence the importance of taste, so regardless of gender, taste remains a critical factor in the decision-making process.

**Figure 1:**
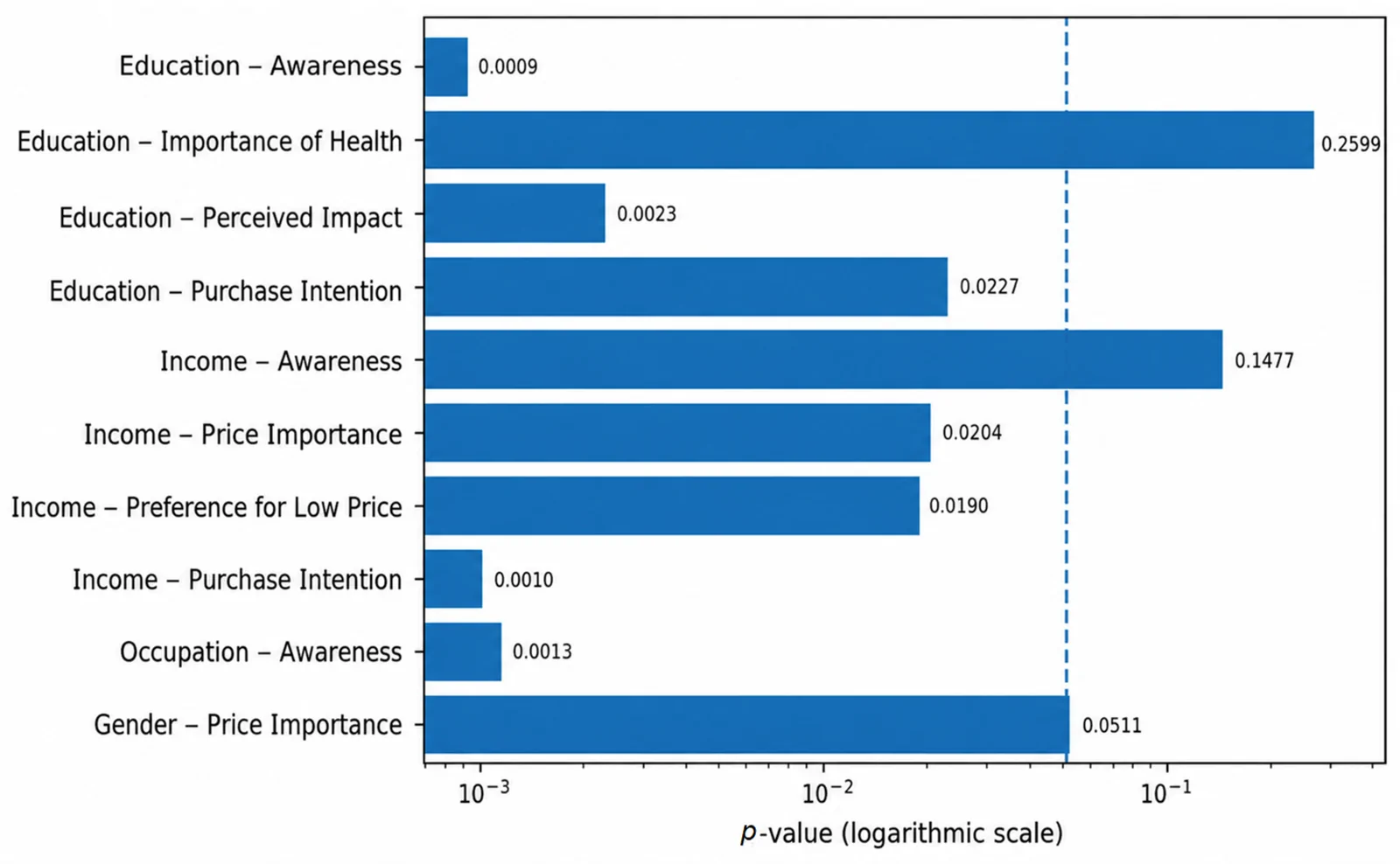
Summary of the statistical significance of the χ^2^ tests (*p* values, logarithmic scale; the threshold of 0.05 is marked by a dashed line). Source: Author’s processing.

Table 8, Table 9 and Table 10 provide the inferential results for education, income, occupation and gender, while Figure 1 offers a compact visual summary of the *p* values for these analyses. To avoid repetition, the tables should be read for the exact χ^2^, df, p and Cramér’s V values, whereas Figure 1 is used only to visualize which associations cross the pre-specified *p* < 0.05 threshold. Overall, the observed associations are weak to moderate rather than strong. Education is associated with awareness, perceived health impact and purchase intention; income is associated with price sensitivity and purchase intention; occupation is associated with awareness; and gender is not significantly associated with price importance. These findings support a multi-determined interpretation of functional-food acceptance within the analyzed sample, but they do not establish independent or causal effects.

Overall, the identified effects are weak to moderate, confirming that the acceptance of functional foods is a multi-determined phenomenon. However, the consistency of significant relationships supports the relevance of education, occupation, and income in explaining differences in perception and behavior within the analyzed sample.

## 4. Discussion

The sample composition is central to the interpretation of the findings. Of the 215 valid respondents, 62.33% were women and 38.14% belonged to Generation Z; the four generations were represented by approximately 82, 52, 43 and 38 respondents, respectively. More than 60% of respondents held at least a bachelor’s degree, and many reported low incomes. These characteristics mean that the findings describe a relatively young, female and comparatively highly educated segment of survey participants rather than the Transylvanian population as a whole. In particular, the observed association between generation and FF awareness should not be interpreted as a pure generational effect, because education, income, occupation and generation may be correlated. The study therefore avoids statements that imply that “Gen Z” or any other cohort is representative of the corresponding population in Transylvania.

The intergenerational interpretation is consistent with Generational Cohort Theory, which argues that consumers who experience similar historical, technological and socio-economic conditions during formative periods may develop distinctive values and consumption patterns. This perspective is useful for functional foods because information sources, trust in scientific claims, health priorities and openness to novel products may differ between cohorts. At the same time, a life-course perspective cautions that apparent generation differences can also reflect age-related changes in health concerns, household structure and economic resources. The Theory of Planned Behavior and the Health Belief Model provide complementary explanations: purchase intention may reflect attitudes and perceived control, while disease-prevention perceptions may reflect perceived susceptibility, severity, benefits and barriers [35,36]. The present cross-sectional design cannot disentangle cohort, age and period effects, so generational differences are treated as descriptive associations rather than causal mechanisms.

In terms of purchasing behavior, the majority of participants (46.51%) are not familiar with the concept of FF, but after reading the explanations, the majority (88.37%) say they have previously purchased functional foods. The research results show that the acceptance of functional foods among consumers in Transylvania is supported primarily by the perceived health benefits, but is limited by an insufficient level of knowledge of the concept. This relationship explains the main peculiarity observed: respondents frequently buy functional products, but do not always recognize them by their specific terminology. Consumption thus precedes conceptual understanding, which confirms that the purchase decision is mainly guided by the practical significance of the product and its association with health.

Taste was rated as very important by 72.09% of respondents, making it a prominent descriptive attribute of the sample. However, the age-related χ^2^ test was not statistically significant (*p* = 0.2543; Cramér’s V = 0.151), so the study does not interpret taste as a generational effect. Rather, the result suggests that taste is broadly valued across respondents, while its relative importance does not differ sufficiently by generation to support an age-based association.

On the situational factors that consumers consider, the data shows that health is the most important, with a majority of 64.19% stating that health is very important in the final purchase decision. In terms of preferences, most participants (41.4%) prefer to consume foods and beverages containing vitamins, but supplements also tend to be important in the market. Regarding trust, the majority of participants believe in the effects of functional foods on the body, with a majority of 86.05% stating that consumption improves overall health and a slightly lower percentage of 78.14% stating that it can prevent disease. The dominant importance attributed to health confirms the role of preventive motivations in the acceptance of functional foods. The results are in line with studies showing that anticipated benefits, trust in the effects of the product and their relevance to one’s well-being influence purchase intention [17,34].

In general, consumers are positive about functional foods. Most of them intend to purchase such products, with 56.74% saying they were very likely to and 30.7% saying they were likely to. Less than 15% of all participants are reluctant towards functional foods. Price remains important, but does not become the central criterion of the decision; its effect depends mainly on income, which shows that economic access can facilitate or limit the transformation of favorable attitudes into actual behavior.

Education was associated with several indicators, including awareness, perceived health impact, purchase intention and the importance attributed to selected product information. However, the observed trend should be interpreted cautiously because the sample contains a relatively high proportion of university-educated respondents and the analysis is bivariate. The result concerning brand importance is particularly important: higher-educated respondents reported greater brand importance in this sample (Cramér’s V = 0.211). This does not imply that lower-educated consumers are generally less reliant on brands; alternative explanations include differences in income, occupation, product exposure, information search and sample composition.

The presence of children can create many influences from different elements, due to the fact that parents tend to be more careful about what they consume in order to protect them. Therefore, people with children are more aware of functional foods. When it comes to health, consumers with children consider them essential factors in the decision-making process. Also, during flu seasons, most childless people tend to take no action to protect themselves, or choose natural supplements. Meanwhile, parents prefer to eat functional foods or at least take natural supplements.

Regardless of generation, gender, and having children or not, taste is a critical factor in the decision-making process for consumers. The importance of brand and packaging is not distinct between people with or without children. Packaging plays a significant role in the decision-making process, but not as critical as the element of taste. Meanwhile, brand and scientific certifications are important to some consumers, but not at the same level as the other elements. Responses related to situational factors show that health plays a critical role in food choice behavior.

The Romanian context gives these findings a more specific public-health relevance. Cardiovascular diseases remain a major health burden in Romania, and national prevention policies emphasize dietary and lifestyle measures [59,60]. The practical implication is not that functional foods should replace medical treatment or a balanced diet, but that evidence-based functional-food communication can be integrated into preventive nutrition. For younger consumers, especially the overrepresented Generation Z group, communication may emphasize convenience, recognizable benefits, product transparency and contemporary food trends without overstating health effects. For Generation Y and X, clear nutritional information, practical health benefits and credible evidence may be more salient. For Baby Boomers, communication can place greater emphasis on disease prevention, scientific certification and transparent evidence. Across all groups, claims must remain consistent with EU requirements for authorized nutrition and health claims.

## 5. Limitations and Future Research Directions

The study has several limitations that constrain interpretation. First, the non-probability sample is not representative of the Transylvanian population: women, younger adults and highly educated respondents are overrepresented, while the Baby Boomer subgroup contains only about 38 respondents. Second, the unequal group sizes reduce the stability of some generational contingency-table comparisons, and apparent cohort differences may be confounded by education, income and occupation. Third, the study relies on bivariate χ^2^ tests and cannot estimate independent effects or interactions through regression or multivariate models. Fourth, the questionnaire is concise and relies partly on single-item indicators; the binary awareness item measures recognition rather than depth of knowledge. Fifth, the study is cross-sectional and cannot separate age, period and cohort effects. Finally, the absence of individual-level raw data prevents more advanced re-analysis and formal model-based adjustment in the present revision.

Future research should use larger, probability-based or quota-controlled samples with balanced representation across generations and socio-economic strata. Individual-level data should be analyzed using multivariable logistic or ordinal regression, with generation, education, income, gender and occupation entered simultaneously and with interaction terms where theoretically justified. Multi-item validated measures of functional-food knowledge, health consciousness, trust and purchase intention should also be used. Cross-country comparisons with Central and Eastern European samples would further clarify which findings are context-specific to Romania and which are shared across European consumer markets.

Future work should also test measurement invariance across generational groups and use longitudinal or repeated cross-sectional designs to distinguish age, period and cohort effects. Where possible, raw individual-level data should be retained so that adjusted effect estimates and uncertainty intervals can be reported.

## 6. Conclusions

In relation to O1, the study shows that respondents in the analyzed sample frequently report purchasing functional foods even when they do not explicitly recognize the FF concept. Taste and packaging are prominent product attributes, while price and purchase intention are associated with income. These findings support H1 only partially: some product-attribute evaluations vary by generation, but taste does not show a statistically significant generational association.

In relation to O2, perceived health benefits are central to acceptance-related attitudes. Most respondents reported confidence that functional foods can improve overall health and help prevent disease. Education and family structure were associated with selected health-related indicators. These findings provide partial support for H2, but the bivariate design does not establish that generation or education independently determines health perceptions.

In relation to O3, generation, education and selected socio-demographic variables were associated with several product-related evaluations. The effects were generally weak to moderate, as indicated by Cramér’s V. H3 is therefore supported as an association hypothesis, not as evidence of causal influence. In particular, the trend observed for the education–brand association should be interpreted as sample-specific and should not be generalized to consumers with lower education.

For Romania, the findings suggest three practical priorities: (1) improve functional-food and nutrition literacy through clear, evidence-based information; (2) ensure that product communication and labeling remain aligned with EU nutrition and health-claim rules; and (3) adapt communication to life-stage and generational priorities without treating cohorts as homogeneous populations. For younger consumers, convenience, transparency and credible trends may improve engagement; for older consumers, disease-prevention information and scientific certification may be more salient. These strategies should complement, rather than replace, balanced diets and appropriate medical care.

The study provides a regional empirical benchmark rather than a population estimate. Its main contribution is the integrated examination of awareness, health perceptions, purchase-related behavior and product attributes across four generational cohorts in a Romanian setting, while explicitly recognizing the limits imposed by non-probability sampling and bivariate analysis.

## Figures and Tables

**Table 1:** The importance of FF attributes on the final purchase decision.

Attribute	Not Important at All	Little Important	Somewhat Important	Important	Very Important
Package	6.98% (95% CI 4.27–11.19%)	18.60% (95% CI 13.97–24.34%)	30.70% (95% CI 24.92–37.16%)	30.23% (95% CI 24.48–36.67%)	13.49% (95% CI 9.56–18.70%)
Taste	0.47% (95% CI 0.08–2.59%)	2.79% (95% CI 1.29–5.95%)	4.65% (95% CI 2.55–8.35%)	20.00% (95% CI 15.20–25.85%)	72.09% (95% CI 65.75–77.66%)
Brand	6.05% (95% CI 3.57–10.07%)	19.53% (95% CI 14.79–25.35%)	35.35% (95% CI 29.27–41.94%)	29.30% (95% CI 23.62–35.71%)	9.77% (95% CI 6.48–14.47%)
Scientific certifications	6.51% (95% CI 3.92–10.63%)	12.56% (95% CI 8.78–17.65%)	26.00% (95% CI 20.64–32.30%)	34.42% (95% CI 28.39–40.99%)	20.51% (95% CI 15.61–26.35%)

Source: Authors’ calculations based on the survey data.

**Table 2:** Detailing the scale of importance.

Degree of Importance	Significance of Interpretation of Results
Not important at all	The attribute never influences the final purchase decision of the functional food.
Little important	The attribute is not that important, so most of the time it is not an influential factor in the purchase decision.
Somewhat important	The attribute can influence the purchase decision, but only sometimes.
Important	Most of the time the attribute presents itself as an influential factor in the purchase and consumption decision.
Very important	The attribute always plays a role in the final purchase decision.

Source: Authors’ calculations based on the survey data.

**Table 3:** Results of the analysis of FF consumption preferences.

	Degree of Preference		
Analysis Parameter	33.95% (95% CI 27.95–40.52%)	24.65% (95% CI 19.37–30.82%)	41.40% (95% CI 35.02–48.07%)
The contribution of FF to the prevention of a mild disease	86.05% (95% CI 80.78–90.05%)	13.95% (95% CI 9.95–19.22%)	0.00%
	78.14% (95% CI 72.15–83.14%)	18.14% (95% CI 13.56–23.83%)	3.72% (95% CI 1.90–7.17%)
The belief that FF can improve overall health	86.05%	13.95%	-
	believes that the consumption of FF can improve overall health	unsure of these health effects	no participant reported complete disbelief
Confidence in the effects of FF on disease prevention	78.14%	18.14%	3.72%
	confident that the consumption of FF can prevent disease	not sure of these beneficial effects	firmly believe that eating FF does not have these effects on the body

Source: Authors’ calculations based on the survey data.

**Table 4:** Socio-demographic structure of the analyzed sample.

	Generation Z 18–25 Years			Generation Y 26–40 Years			Generation X 41–55 Years				Baby Boomers 56–76 Years			
Age	38.14% (95% CI 31.91–44.79%)			24.19% (95% CI 18.95–30.33%)			20.00% (95% CI 15.20–25.85%)				17.67% (95% CI 13.16–23.33%)			
Gender	**Female**						**Male**							
	62.33% (95% CI 55.69–68.53%)						37.67% (95% CI 31.47–44.31%)							
Family with kids	**No kids**						**At least 1 kid**							
	54.42% (95% CI 47.74–60.94%)						45.58% (95% CI 39.06–52.26%)							
Education level	**Secondary school**		**Vocational or technical school**		**High school**		**Bachelor’s degree**		**Post-graduate**		**Masters**		**PhD**	
	2.33% (95% CI 1.00–5.33%)		4.19% (95% CI 2.22–7.76%)		25.58% (95% CI 20.21–31.81%)		26.98% (95% CI 21.49–33.28%)		6.51% (95% CI 3.92–10.63%)		20.47% (95% CI 15.61–26.35%)		13.95% (95% CI 9.95–19.22%)	

Source: Authors’ calculations based on the survey data.

**Table 5:** Chi-Square test of the influence of age on FF analysis parameters.

Parameter	χ^2^	df	*p* Value	Cramér V	Interpretation Result
Awareness	19.140	6	0.0039	0.211	At a 95% confidence level, age definitely influences awareness of FF concept.
Package	29.536	12	0.0033	0.214	With a 95% confidence level age influences packaging. All generations, regardless of age, pay attention to packaging.
Taste	14.769	12	0.2543	0.151	Regardless of age, taste is a critical factor in the decision-making process.
Certifications	31.320	12	0.0018	0.220	Certifications are important to all participants. For each older generation, this component becomes more important, due to the trend towards healthier consumption as they age.
Nutritional information	38.027	12	0.0002	0.243	Generation Z does not place much importance on this component, and Baby Boomers do not consider it as much as Generation X and Y read relevant information before making a final decision.
Preference for consumption during periods of flu	20.146	6	0.0026	0.216	Gen Z are more inclined to supplement or do nothing rather than functional foods during flu season. The other generations tend to consume functional foods and trust the health benefits of their food choices.

Source: Authors’ calculations based on the survey data.

**Table 6:** Chi-Square test of the influence of education on FF analysis parameters.

Parameter	χ^2^	df	*p* Value	Cramér V	Interpretation Result
Awareness	33.263	12	0.0009	0.278	With a 95% confidence level, education influences awareness of the concept of FF.
Package	41.011	24	0.0166	0.218	Education influences the type of packaging.
Brand	38.331	24	0.0321	0.211	Consumers with a lower level of education are not as influenced by the brand as those with a higher level of education.
Certifications	36.780	24	0.0460	0.207	Consumers with higher levels of education consider the presence of certifications more important.
Impact on health	30.519	12	0.0023	0.266	Participants with higher levels of education have more confidence in functional products, with small differences between those with master’s and doctoral degrees.
Perception and confidence to prevent disease	22.764	12	0.0298	0.230	With a 95% confidence level, education influences consumer perception and trust.
Preference for consumption during periods of flu	15.209	12	0.2302	0.188	Education does not influence what participants consume during flu seasons, so regardless of education level, consumer preferences do not change.
Nutritional information	37.877	24	0.0357	0.210	Education influences the importance of nutrition information and all participants consider it important, but those with higher levels of education tend to be more influenced.

Source: Authors’ calculations based on the survey data.

**Table 7:** Chi-Square test of the influence of family structure and gender on the analysis parameters of FF.

Parameter	χ^2^	df	*p* Value	Cramér V	Interpretation Result
Awareness	13.632	2	0.0011	0.252	With a 95% confidence level, family structure influences consumer awareness of the concept. Families with children know the concept of FF better than those without children.
Package	5.831	4	0.2122	0.116	Family structure does not influence the importance of packaging. Families with or without children put the same emphasis on packaging, which is important but not critical in the final purchase decision.
Taste	2.235	4	0.6926	0.072	Family structure does not influence the importance of taste, so the presence of children in a family does not change the importance that taste has for the final purchase decision.
Brand	8.786	4	0.0667	0.143	With a confidence level of 95%, family structure does not influence brand importance. Children do not influence their parents to buy products with well-known brands.
Health component of the products	23.262	4	0.0001	0.233	Family structure influences the importance of this component. Families with children consider health more important than families without children.
Preference in consumption during flu periods.	13.665	2	0.0011	0.252	Family structure influences preferences. Families with children consume more functional foods to boost their immune systems, and families without children take fewer steps and tend to do nothing to protect themselves.
The influence of gender on taste	6.085	4	0.1929	0.168	Gender does not influence the importance of taste, so regardless of gender, taste remains a critical factor in the decision-making process.

Source: Authors’ calculations based on the survey data.

**Table 8:** Association between education and FF parameters.

Parameter	Statistics (χ^2^)	Df	*p* Value	Cramér V	Conclusion
Awareness	33,263	12	0.0009	0.28	Significant association
Importance of health	27,769	24	0.2699	0.18	No significant association
Perceive health impact	30,519	12	0.0023	0.27	Significant association
Purchase intention	39,756	24	0.0227	0.21	Significant association

Source: Authors’ calculations based on the survey data.

**Table 9:** Association between income and FF parameters.

Parameter	Statistics (χ^2^)	Df	*p* Value	Cramér V	Conclusion
Awareness	12,079	8	0.1477	0.17	No significant association
Price importance	29,570	16	0.0204	0.19	Significant association
Preference for low price	29,807	16	0.0190	0.19	Significant association
Purchase intention	39,376	16	0.0010	0.21	Significant association

Source: Authors’ calculations based on the survey data.

**Table 10:** Association with profession (occupation) and gender.

Parameter	χ^2^	Df	*p* Value	Cramér V	Conclusion
Profession (Occupation)—Awareness	25,409	8	0.0013	0.24	Significant association
Gender—Price importance	9434	4	0.0511	0.21	No significant association

Source: Authors’ calculations based on the survey data.

## Data Availability

The data presented in this study is available on request from the corresponding author. The data is not publicly available due to privacy reasons.

## Abbreviations

The following abbreviations are used in this manuscript:

FF	Functional Foods

## Appendix A. Questionnaire Structure

Q1. Are you familiar with the concept of functional food? [Yes/No]

Q2. Have you already purchased any functional foods? [Yes/No]

Q3. Where do you buy groceries? [Supermarket/City market (grocery market)/Specialized store (e.g., Bonas)/Directly from the producer/Others]

Q4. When buying groceries, how important are the following elements (Packaging/Taste/Price/Brand/Certifications/Nutritional information/Durability (shelf-life))? [Not important at all/Not that important/Somehow important/Important/Very important]

Q5. From which categories do you acquire functional products? [Dairy/Fruits and vegetables/Tea/Cereals/Meat/Others/I do not buy any functional products]

Q6. When buying foods and beverages, how important are the following factors (Health/Cheap price/Fun/Trend/Convenience) in your final decision? [Not important at all/Not that important/Somehow important/Important/Very important]

Q7. During flu periods, what do you consume? [Supplements (e.g., vitamin C, D, pollen, Omega-3 etc.)/Food and beverage products that contain vitamins/I am just careful and do nothing specific for preventing getting sick]

Q8. Do you believe that consuming functional foods can improve your general health? [Yes/I do not know/No]

Q9. Do you believe that consuming functional foods can prevent various illnesses (e.g., diabetes, cardiovascular diseases etc.)? [Yes/I do not know/No]

Q10. Do you intend to buy functional products in the future? [It is very unlikely/It is unlikely/Not sure/It is likely/It is very likely]

Q11. What is your age? [18,19,20,21,22,23,24,25,26,27,28,29,30,31,32,33,34,35,36,37,38,39,40,41,42,43,44,45,46,47,48,49,50,51,52,53,54,55,56,57,58,59,60,61,62,63,64,65,66,67,68,69,70,71,72,73,74,75,76]

Q12. What is your gender? [Female/Male]

Q13. Please indicate the highest level of education completed [Gymnasium/High school/Vocational or Technical School/Postgraduate Studies/Bachelor Studies/Master Studies/PhD Studies]

Q14. What is your professional activity? [Student/Employed/Unemployed/Self-employed/Retired]

Q15. What is your income? [0–1000 lei/1000–2500 lei/2500–3500 lei/3500–4500 lei/>4500 lei]

Q16. What is your social status? [Single/In a relationship/Married/Divorced/Widowed]

Q17. Do you have children? [Yes/No]

Note: The manuscript documents the 14-question structure and response categories used in the reported analyses. If the journal requires verbatim reproduction of the original Google Forms instrument, the original form/export should replace this structured appendix before final submission.

## References

[1] Gibson G., Williams C. (2003). Functional Foods.

[2] Anton D.T., Coman A.E. (2011). Role of Functional Foods in Health Promotion in Children.

[3] Betoret E., Betoret N., Vidal D., Fito P. (2011). Functional foods development: Trends and technologies. Trends Food Sci. Technol..

[4] Khan R.S., Grigor J., Winger R., Win A. (2013). Functional food product development—Opportunities and challenges for food manufacturers. Trends Food Sci. Technol..

[5] Gök I. (2023). Functional potential of several Turkish fermented traditional foods: Biotic properties, bioactive compounds, and health benefits. Food Rev. Int..

[6] Frumuzachi O., Rohn S., Mocan A. (2024). Fermented black chokeberry (*Aronia melanocarpa* (Michx.) Elliott)—A systematic review of composition and current scientific evidence on possible health benefits. Food Res. Int..

[7] Kalita P., Chakrabarti S., Bhattacharjee B., Paul S., Dutta P.P., Pachuau L. (2025). Recent advances in improving the delivery, bioavailability and bioactivity of polyphenolic compounds through encapsulation: A comprehensive review. Food Chem..

[8] (1999). Scientific Concepts of Functional Foods in Europe Consensus Document. Br. J. Nutr..

[9] Van de Kaa E.J. (2010). Prospect Theory and Choice Behaviour Strategies: Review and Synthesis of Concepts from Social and Transport Sciences. Eur. J. Trans. Inf. Res..

[10] Brătianu C. (2015). Chapter 5. The trust. Strategic Thinking.

[11] Arai S. (2002). Global view on functional foods: Asian perspectives. Br. J. Nutr..

[12] Voidarou C., Antoniadou M., Rozos G., Tzora A., Skoufos I., Varzakas T., Lagiou A., Bezirtzoglou E. (2021). Fermentative foods: Microbiology, biochemistry, potential human health benefits and public health issues. Foods.

[13] Rahman M.M., Dhar P.S., Sumaia Anika F., Ahmed L., Islam M.R., Sultana N.A., Cavalu S., Pop O., Rauf A. (2022). Exploring the plant-derived bioactive substances as antidiabetic agent: An extensive review. Biomed. Pharmacother..

[14] Verbeke W. (2005). Consumer acceptance of functional foods: Sociodemographic, cognitive and attitudinal developments. Food Qual. Prefer..

[15] Urala N., Lahteenmaki L. (2007). Consumers changing attitudes towards functional foods. Food Qual. Prefer..

[16] Siegrist M., Stampfli N., Kastenholz H. (2008). Consumers’ willingness to buy functional foods. The influence of carrier, benefit and trust. Appetite.

[17] Huang L., Bai L., Zhang X., Gong S. (2019). Re-understanding the antecedents of functional foods purchase: Mediating effect of purchase attitude and moderating effect of food neophobia. Food Qual. Prefer..

[18] Ravoniarison A. (2017). Senior consumers and risk/benefit trade-off in functional foods. Br. Food J..

[19] Bech-Larsen T., Scholderer J. (2007). Functional foods in Europe: Consumer research, market experiences and regulatory aspects. Trends Food Sci. Technol..

[20] Vicentini A., Liberatore L., Mastrocola D. (2016). Functional Foods: Trends and Development of the Global Market. Ital. J. Food Sci..

[21] Castellini A., Canavari M., Pirazzoli C. Functional foods in the European Union: An overview of the sector’s main issues. Proceedings of the 8th Joint Conference on Food, Agriculture and the Environment.

[22] Bagchi D., Nair S. (2017). Development of New Functional Food and Nutraceutical Products.

[23] Fuller G.W. (2016). New Food Product Development: From Concept to Marketplace.

[24] Gilbert L. (1998). The consumer market for functional foods. J. Nutr. Func. Med. Foods.

[25] Mark-Herbert C. (2004). The innovation of a new product category—Functional foods. Technovation.

[26] Williams M., Pehu E., Ragasa C. (2006). Functional Foods: Opportunities and Challenges for Developing Countries. Agricultural and Rural Development Notes.

[27] Siro I., Kapolna E., Kapolna B., Lugasi A. (2008). Functional food. Product development, marketing and consumer acceptance—A review. Appetite.

[28] Kotilainen L., Rajalahti R., Ragasa C., Pehu E. (2006). Health enhancing foods: Opportunities for strengthening the sector in developing countries. Agriculture and Rural Development Discussion Paper 30.

[29] Lalor F., Madden C., Mckenzie K., Wall P.G. (2011). Health claims on foodstuffs: A focus group study of consumer attitudes. J. Funct. Foods.

[30] Bhaskaran S., Hardley F. (2002). Buyer beliefs, attitudes and behavior: Foods with therapeutic claims. J. Consum. Mark..

[31] Chen M.F. (2011). The joint moderating effect of health consciousness and healthy lifestyle on consumers’ willingness to use functional foods in Taiwan. Appetite.

[32] Aschemann-Witzel J., Zielke S. (2017). Can’t buy me green? A review of consumer perceptions of and behavior toward the price of organic food. J. Consum. Aff..

[33] Koen N., Wentzel-Viljoen E., Blaauw R. (2018). Price rather than nutrition information the main influencer of consumer food purchasing behaviour in South Africa: A qualitative study. Int. J. Consum. Stud..

[34] Horská E., Predanócyová K., Šedík P., Grunert K.G., Hupková D. (2023). Consumer perception of functional foods and determinants of functional food consumption in the Slovak Republic. Br. Food J..

[35] Ajzen I. (1991). The theory of planned behavior. Organ. Behav. Hum. Decis. Process.

[36] Rosenstock I.M. (1974). Historical origins of the Health Belief Model. Health Educ. Monogr..

[37] Dean M., Lampila P., Shepherd R., Arvola A., Saba A., Vassallo M., Claupein E., Winkelmann M., Lähteenmäki L. (2012). Perceived relevance and foods with health-related claims. Food Qual. Prefer..

[38] Cabal-Prieto A., Herrera-Corredor J.A., Vega-Carreño M.I., Chay-Canul A.J., Chareo-Benítez B., Juarez-Barrientos J.M., Hernández-Salinas G., Guerrero-Ortíz C.A., Armida-Lozano J., Ramírez-Rivera E.J. (2024). Analysis of sensory and cognitive performance of generational consumers using artisan tortillas. J. Sens. Stud..

[39] Uliano A., Lerro M. (2025). Generational Preferences and Willingness to Pay for Antioxidant-Rich Pomegranates: Insights into Consumer Behavior and Market Potential. Agriculture.

[40] Kumar N., Kapoor S. (2017). Do labels influence purchase decisions of food products? Study of young consumers of an emerging market. Brit Food J..

[41] Ares G., Gambaro A. (2007). Influence of gender, age and motives underlying food choice on perceived healthiness and willingness to try functional foods. Appetite.

[42] Hanspal S., Devasagayam P.R. (2017). Impact of Consumers’ Self-Image and Demographics on Preference for Healthy Labeled Foods. Sage Open.

[43] Gould S.J. (1988). Consumer attitudes toward health and health care: A differential perspective. J. Consum. Aff..

[44] Michaelidou N., Hassan L. (2008). The role of health consciousness, food safety concern and ethical self-identity on attitudes and intentions towards organic food. Int. J. Consum. Stud..

[45] Puhakka R., Valve R., Sinkkonen A. (2017). Older consumers’ perceptions of functional foods and non-edible health-enhancing innovations. Int. J. Consum. Stud..

[46] Landstrom E., Koivisto Hursti U.-K., Becker W., Magnusson M. (2007). Use of functional foods among Swedish consumers is related to health-consciousness and perceived effect. Br. J. Nutr..

[47] Niva M. (2006). Can we predict who adopts health-promoting foods? Users of FF in Finland. Scand. J. Food Nutr..

[48] Barreiro-Hurlé J., Colombo S., Cantos-Villar E. (2008). Is there a market for functional wines? Consumer preferences and willingness to pay for resveratrol-enriched red wine. Food Qual. Prefer..

[49] Childs N.M. (1997). Functional Foods and the Food Industry. J. Nutr. Func. Med. Foods.

[50] Wrick K.L. (1995). Consumer issues and expectations for functional foods. Crit. Rev. Food Sci. Nutr..

[51] Niva M. Consumers, functional foods and everyday knowledge. Proceedings of the Conference of Nutritionists Meet Food Scientists and Technologists.

[52] Falguera V., Aliguer N., Falguera M. (2012). An integrated approach to current trends in food consumption: Moving toward functional and organic products?. Food Control.

[53] Hilliam M. (1996). Functional foods: The Western consumer viewpoint. Nutr. Rev..

[54] Pop N.A., Dabija D.-C. (2013). Development of an organic food market in Romania. Amfiteatru Econ..

[55] Petrescu D.C., Petrescu-Mag R.M. (2015). Organic food perception: Fad, or healthy and environmentally friendly? A case on Romanian consumers. Sustainability.

[56] Oroian C.F., Safirescu C.O., Harun R., Chiciudean G.O., Arion F.H., Mureșan I.C., Bordeanu B.M. (2017). Consumers’ attitudes towards organic products and sustainable development: A case study of Romania. Sustainability.

[57] Voinea L., Vrânceanu D.M., Filip A., Popescu D.V., Negrea T.M., Dina R. (2019). Research on food behavior in Romania from the perspective of supporting healthy eating habits. Sustainability.

[58] Precup G., Pocol C.B., Teleky B.-E., Vodnar D.C. (2022). Awareness, knowledge, and interest about prebiotics—A study among Romanian consumers. Int. J. Environ. Res. Public Health.

[59] National Institute of Public Health (2024). Situation Analysis—Cardiovascular Diseases (Data for 2023). National Campaign for the Prevention of Cardiovascular Diseases. Bucharest. https://insp.gov.ro.

[60] Government of Romania (2025). National Strategy for Combating Cardiovascular and Cerebrovascular Diseases 2025–2030.

[61] European Parliament and Council (2006). Regulation (EC) No 1924/2006 on Nutrition and Health Claims Made on Foods. Official Journal of the European Union. https://eur-lex.europa.eu/eli/reg/2006/1924/oj/eng.

[62] European Parliament and Council (2015). Regulation (EU) 2015/2283 on Novel Foods. Official Journal of the European Union. https://eur-lex.europa.eu/eli/reg/2015/2283/oj/eng.

[63] European Parliament and Council (2011). Regulation (EU) No 1169/2011 on the Provision of Food Information to Consumers. Official Journal of the European Union. https://eur-lex.europa.eu/eli/reg/2011/1169/oj/eng.

[64] Trigui I.T., Giraud G. (2012). Brand Proneness versus experiential effect? Explaining consumer preferences towards labeled food products. J. Glob. Sch. Mark. Sci..

[65] Mohd Daud N., Ramli L., Jemahdi N., Husna Razalli R. (2011). Examining critical success factors of consumers’ attitude towards nutritional labelling of SMEs products in Malaysia. Aust. J. Basic Appl. Sci..

[66] Lagerkvist C.J. (2013). Consumer preference for food labelling attributes: Comparing direct ranking and best-worst scaling for measurement of attribute importance, preference intensity and attribute dominance. Food Qual. Prefer..

[67] Van Boxsteal S., Devlieghere F., Berkvens D., Vermeulen A., Uyttendaele M. (2014). Understanding and attitude regarding the shelf life labels and dates on prepackaged food products by Belgian consumers. Food Control.

[68] Steinhauser J., Janssen M., Hamm U. (2019). Consumers’ purchase decisions for products with nutrition and health claims: What role do product category and gaze duration on claims play?. Appetite.

[69] Belei N., Geyskens K., Goukens C., Ramanathan S., Lemmink J. (2012). The best of both worlds? Effects of attribute-induced goal conflict on consumption of healthful indulgences. J. Mark. Res..

[70] Gravel K., Doucet É., Peter Herman C., Pomerleau S., Bourlaud A.-S., Provencher V. (2012). “Healthy,” “diet,” or “hedonic”. How nutrition claims affect food-related perceptions and intake?. Appetite.

[71] Larkin D., Martin C.R. (2016). Caloric estimation of healthy and unhealthy foods in normal-weight, overweight and obese participants. Eat. Behav..

[72] Orquin J.L., Scholderer J. (2015). Consumer judgments of explicit and implied health claims on foods: Misguided but not misled. Food Policy.

[73] Otterbring T., Gidlöf K., Rolschau K., Shams P. (2020). Cereal Deal: How the Physical Appearance of Others Affects Attention to Healthy Foods. Perspect. Behav. Sci..

[74] Vila-López N., Küster-Boluda I., Sarabia-Sánchez F. (2017). Designing a packaging to promote healthy and low-fat foods: Adolescents versus young-adults. Food Res. Int..

[75] Küster I., Vila N. (2017). Health/Nutrition food claims and low-fat food purchase: Projected personality influence in young consumers. J. Funct. Foods.

[76] Taghavi M.-S., Seyedsalehi A. (2015). The effect of packaging and brand on children’s and parents’ purchasing decisions and the moderating role of pester power. Brit Food J..

[77] Rettie R., Brewer C. (2000). The verbal and visual components of package design. J. Prod. Brand Manag..

[78] Prendergast P.G., Pitt L. (1996). Packaging, marketing, logistics and the environment: Are there trade-offs?. Int. J. Phys. Distrib. Log..

[79] Ferreira B.M. (2019). Packaging texture influences product taste and consumer satisfaction. J. Sens. Stud..

[80] Gelici-Zeko M.M., Lutters D., Klooster R.T., Weijzen P.L.G. (2012). Studying the Influence of Packaging Design on Consumer Perceptions (of Dairy Products) Using Categorizing and Perceptual Mapping. Packag. Technol. Sci..

[81] Vella M.N., Stratton L.M., Sheeshka J., Duncan A.M. (2013). Exploration of functional food consumption in older adults in relation to food matrices, bioactive ingredients, and health. J. Nutr. Gerontol. Geriatr..

[82] Van Kleef E., Van Trijp H.C.M., Luning P. (2005). Functional foods: Health claim-food product compatibility and the impact of health claim framing on consumer evaluation. Appetite.

[83] Rybanská J., Nagyová Ľ. (2017). The sense of taste. Sensory and Aroma Marketing.

[84] Bratanova B., Kervyn N., Klein O. (2015). Tasteful Brands: Products of Brands Perceived to be Warm and Competent Taste Subjectively Better. Psychol. Belg..

[85] Glanz K., Basil M., Maibach E., Goldberg J., Snyder D.A.N. (1998). Why Americans eat what they do: Taste, nutrition, cost, convenience, and weight control concerns as influences on food consumption. J. Am. Diet. Assoc..

[86] Shepherd R., Sparks P., MacFie H., Thomson D. (1994). Modelling food choice. Measurement of Food Preferences.

[87] De Jong N., Ocke M.C., Branderhorst H.A.C., Friele R. (2003). Demographic and lifestyle characteristics of functional food consumers and dietary supplement users. Brit J. Nutr..

[88] Pascal G. (2009). Functional Foods in the European Union. Nutr. Rev..

[89] Foxall G.R., Yan J., Oliveira-Castro J.M., Wells V.K. (2013). Brand-related and situational influences on demand elasticity. J. Bus. Res..

[90] Pieters R., Warlop L. (1999). Visual attention during brand choice: The impact of time pressure and task motivation. Int. J. Res. Mark..

[91] Iyer P.P., Paswan A.K., Davari A. (2016). Brands, love and family. J. Prod. Brand Manag..

[92] Morais C.D., Afonso C., Almeida M.D. (2010). Ageing and food consumption in Portugal: New or old paradigms?. Brit Food J..

[93] Sudbury L., Simcock P. (2009). A multivariate segmentation model of senior consumers. J. Consum. Mark..

[94] Van Der Zanden L.D.T., Van Kleef E., De Wijk R.A., Van Trijp H.C.M. (2015). Examining heterogeneity in elderly consumers’ acceptance of carriers for protein-enriched food: A segmentation study. Food Qual. Prefer..

[95] Yoon C., Cole C.A., Haugtvedt C.P., Herr P.M., Kardes F.R. (2008). Aging and consumer behavior. Handbook of Consumer Psychology.

[96] Herath D., Cranfield J., Henson S. (2008). Who consumes functional foods and nutraceuticals in Canada? Results of cluster analysis of the 2006 survey of Canadians’ demand for food products supporting health and wellness. Appetite.

[97] Krystallis A., Maglaras G., Mamalis S. (2008). Motivations and cognitive structures of consumers in their purchasing of functional foods. Food Qual. Prefer..

[98] Sloan A.E. (2005). Demographic directions: Mixing up the market- state of the industry report. Food Technol..

[99] Richards A., Kattelmann K.K., Ren C. (2006). Motivating 18 to 24 years-old to increase their fruit and vegetable consumption. J. Am. Diet. Assoc..

[100] Kamenidou I.E., Stavrianea A., Bara E.Z. (2020). Generational Differences toward Organic Food Behavior: Insights from Five Generational Cohorts. Sustainability.

[101] Juscius V., Viskantaite I. (2010). Consumer behavior models for Internet marketing. Manag. Theor. Stud. Rural. Bus. Dev..

[102] Zitkus L., Puskoriute N. (2013). Consumer behaviour and its influence on consumer rights violations. Eur. Integr. Stud..

[103] Kushwaha G.S., Sharma N.K. (2017). Factors Influencing Young Entrepreneurial Aspirant’s Insight Towards Sustainable Entrepreneurship. Iran. J. Manag. Stud..

[104] Kolstad I., Wiig A. (2015). Education and entrepreneurial success. Small Bus. Econ..

[105] Hessels J., van Gelderen M., Thurik R. (2008). Entrepreneurial motivations, aspirations and their drivers. Small Bus. Econ..

[106] Cohen J. (1988). Statistical Power Analysis for the Behavioral Sciences.

